# Impact of Hepatitis C Virus Infection on Bone Mineral Density in Renal Transplant Recipients

**DOI:** 10.1371/journal.pone.0063263

**Published:** 2013-05-13

**Authors:** Wen-Hung Huang, Mei-Ching Yu, Jeng-Yi Huang, Ping-Chin Lai

**Affiliations:** 1 Department of Nephrology, Chang Gung Memorial Hospital, Linkou, Taiwan, R.O.C; 2 Department of Paediatric Nephrology, Chang Gung Children’s Hospital, Linkou, Taiwan, R.O.C; 3 Chang Gung University College of Medicine, Taoyuan, Taiwan, R.O.C; Inserm, U1052, UMR 5286, France

## Abstract

**Background:**

The average prevalence of hepatitis C virus (HCV) infection in renal transplant recipients is 10%. Studies of these patients with HCV infection usually focuses on long-term graft survival and patient survival. Studies of the correlation between HCV infection and bone mineral density (BMD) in renal transplant patients are limited. The aim of this study was to investigate whether HCV infection is a risk factor for BMD change during a short follow-up period.

**Methods:**

Seventy-six renal transplant recipients underwent 2 separate dual-energy X-ray absorptiometry (DXA) scans during a mean period of 14 months. Fifteen patients were HCV infection. First bone mineral density (BMD) at the lumbar spine, hip, and femoral neck was determined using dual-energy X-ray absorptiometry (DXA) between September 2008 and March 2009. After that, 34 patients took alendronate sodium 70 mg per week. Subgroups risk factors analysis was also performed into with or without alendronate. Immunosuppressive agents, bisphosphonates, patient characteristics, and biochemical factors were analyzed to identify associations with BMD.

**Results:**

After 14 months, in 76 patients, BMD of the lumbar spine had significantly increased (from 0.9 g/cm^2^ to 0.92 g/cm^2^, p<0.001), whereas BMD of the hip and femoral neck had not. Multiple linear regression analysis showed that HCV infection was negatively associated with BMD change in the lumbar spine (***β*** *:* −0.247, 95% CI, −0.035 to −0.002; *p* = 0.028). Moreover, in subgroup analysis, among 42 patients without alendronate, multiple linear regression analysis showed HCV infection was a risk factor for adverse BMD change of the lumbar spine (***β***: −0.371, 95% CI, −0.043 to −0.003; *p* = 0.023).

**Conclusion:**

HCV infection in renal transplant recipients was a negative risk factor for BMD change in the lumbar spine. Moreover, alendronate may be able to reverse the negative effect of HCV infection on bone in renal transplant recipients.

## Introduction

The natural history of hepatitis C virus (HCV) infection after renal transplantation is not fully understood [1]. HCV infection is still highly prevalent among patients with end-stage renal disease (ESRD), including renal transplant (RT) recipients, despite the screening of blood products for HCV [1], [2]. The prevalence of HCV infection in RT recipients ranges from 5% to 15% [3], [4]. Kidney transplantation in the presence of HCV infection has adverse effects on long-term graft survival and patient survival [5]–[7]. In the general population, lower bone mineral density (BMD) has been associated with chronic liver disease, including chronic viral hepatitis [8], and the pathophysiological basis of this association remains unclear. It may include alterations in vitamin D metabolism, gonadal status, or chronic inflammation. A direct role for chronic HCV infection in the pathogenesis of osteoporosis is further supported by the observation that fracture risk decreased in HCV-monoinfected patients who underwent successful antiviral treatment [9]. In a study of patients infected with either hepatitis B or C, but not with HIV, the severity of osteoporosis was related to the severity of liver disease by concomitant histology, even among those without the evidence of cirrhosis [10]. However, HCV co-infection has been identified as an independent risk factor for subsequent fragility fracture in multiple cohorts of HIV-infected persons [11], [12]. In previous studies [13], [14], we found that renal transplant recipients with HCV infection had a higher prevalence of osteoporosis than patients without HCV infection. After a long period of renal transplantation, the detrimental effects of steroid and immunosuppressive agents on bone condition diminished. Short-term alendronate administration effectively improves BMD in these patients. To our knowledge, studies of the correlation between HCV infection and BMD in renal transplant patients are few. And the role of HCV infection on BMD in renal transplant patients with or without alendronate is unclear. The aim of this observational case-control study was to investigate whether HCV infection is a risk factor for BMD change during a short follow-up period.

## Materials and Methods

This retrospective case-control study complied with the guidelines of the Declaration of Helsinki and was approved by the Medical Ethics Committee of Chang Gung Memorial Hospital, a tertiary referral center located in the northern part of Taiwan. Institutional Review Board approval was obtained, and because the study involved retrospective review of existing data, informed consent from the patients was not required. In addition, all individuals’ information was securely protected (by delinking identifying information from the main data set) and available to investigators only. Furthermore, all the data were analyzed anonymously. On the other hand, if this study had involved retrospective review of existing data in addition to retrospective analysis of remaining biological samples, both Institutional Review Board approval and specific informed consent from all patients would have been required. The Institutional Review Board of Chang Gung Memorial Hospital has specifically waived the requirement for consent. All primary data were collected according to Strengthening the Reporting of Observational Studies in Epidemiology guidelines [15]. The form described above was referenced from the previous study [14] and Liu et al.’s publication [16].

### Study Sample

In this retrospective designed study, initially, we randomly enrolled 84 patients undergone transplant at least 5 months previously and had received DXA to obtain BMD measurements of the lumbar spine (LS), left hip (H), and femoral neck (FN) between September 2008 and March 2009 [13]. Alendronate (alendronate sodium, 70 mg/tablet, 70 mg per week) was administered to the patients until osteoporosis was diagnosed. Bone condition was defined on the basis of the World Health Organization (WHO) criteria: a BMD value >2.5 standard deviations (SD, T score) below the mean for young adults indicated osteoporosis, and a BMD value between 1.0 and 2.5 SDs below the mean indicated osteopenia. In previous study [14], use of alendronate was found to be associated with positive prognosis of bone condition in renal transplantation patients. From above conception, the patients were divided into two groups: with or without alendronate for subgroup analysis. Fasting blood levels of serum creatinine (Cr), blood urea nitrogen (BUN), calcium, inorganic phosphate, and uric acid were evaluated. The corrected calcium (C-calcium) concentration was calculated using the following equation: C-calcium = serum calcium (mg/dL)+[0.8 (4.0 - serum albumin (g/dL)]. The patients’ medical records were assessed for history of diabetes mellitus (DM); smoking frequency; alcohol intake; and hepatitis B virus (HBV), hepatitis C virus (HCV), and cytomegalovirus (CMV) infections. Then, 76 of the 84 patients (8 of the 84 patients were excluded–2 subjects had died, 2 had graft failure, and the initial BMD measurements of 4 patients were lost) received 2^nd^ DXA to obtain BMD measurements and fasting blood tests after a mean period of 14 months. All doses of an immunosuppressive agent administered between the 2 BMD measurements were considered as the cumulative dose.

### Precautions and Contraindications for Alendronate

The first DXA report was obtained between September 2008 and March 2009. Alendronate (70 mg per week) was administered to the patients with osteoporosis diagnosed based on this DXA report, unless 1 or more of the following conditions was present: bisphosphonate allergy; blood calcium <8 mg/dL; active stomach problems (e.g., esophagitis, gastritis, or ulcers); renal insufficiency (estimated glomerular filtration rate [eGFR] <30 mL/[min·1.73 m^2^] or serum Cr >3 mg/dL); difficulty in swallowing or inability to stand/sit upright for at least 30 min; and pregnancy or breastfeeding. The patients did not receive alendronate before the first DXA results were obtained. The patients were instructed to take the drug only upon getting up for the day, with 3–4 swallows of water; then fast for 30–45 min while standing, sitting, or walking; and finally eat breakfast. They were not to lie down or recline after taking the drug. Supplements or other drugs should be taken no sooner than 30 min after alendronate.

### Immunosuppression Protocol

Our hospital primarily uses a calcineurin inhibitor (CI)–based immunosuppressive regimen in the initial months after transplantation. Most of our patients also receive mycophenolic acid plus prednisolone during this stage. Immediately after transplantation, the targeted cyclosporine concentration at 2 h post-dose (C_2_) is approximately 1300–1100 ng/mL, and the tacrolimus trough level is maintained at approximately 12–10 ng/mL. These concentrations are gradually tapered in the first year. In patients that have had a transplant for more than 12 months, the cyclosporine C_2_ level is maintained at approximately 500–600 ng/mL and the tacrolimus level at 3–4 ng/mL. Prednisolone is maintained at 1.25–10.00 mg per day, according to the patient’s condition. An mTOR inhibitor is added to the regimen if the patient’s condition is suitable (proteinuria <800 mg/day and eGFR >40 mL/[min·1.73 m^2^]). The trough level of the mTOR inhibitor is maintained at approximately 3–8 ng/mL. Once the mTOR inhibitor has been added the CI and mycophenolic acid doses are cut by 50% overnight, while prednisolone is maintained at the same dosage. Subsequently, the CI dose is tapered as much as possible. Most of the patients in this study received only 25 mg cyclosporine or 0.5 mg tacrolimus per day if an mTOR inhibitor was used.

### Statistical Analysis

Values of variables with non-normal distributions are presented as medians and interquartile ranges; data for variables with normal distributions are expressed as means ± SDs. Paired *t* test and Wilcoxon signed rank test were used to compare baseline and follow-up data. Groups were compared using the Mann–Whitney test and *t* test. Initially, simple linear regression analysis was used to identify immunosuppressive agents and biochemical and demographic variables associated with BMD change. The following factors were investigated: age, sex, time since transplantation, age at transplantation, use of prednisolone, use of mycophenolate, use of tacrolimus, use of sirolimus, use of cyclosporine, smoking habits, alcohol consumption, HBV infection, HCV infection, and CMV infection. Multiple linear regression analysis (stepwise forward approach) was then applied to assess predictors of BMD change. To calculate the relative risk of BMD change, standardized coefficients (***β***) and 95% confidence intervals (CIs) were obtained by linear regression analysis. All the nominal variables in linear regression were dummy coding transformed.The chi-squared test was used to determine the correlation between 2 binary variables; a *p* value <0.05 was considered statistically significant. All statistical analyses were performed using the Statistical Package for the Social Sciences (SPSS), version 12.0 for Windows (SPSS, Inc.; Chicago, IL, USA). Because of the exploratory nature of the study and the consequent lack of priori knowledge about effective size, no formal sample size computations were performed. F test was used to calculate post hoc power in this study. In the study of 76 patients of sample size, an effective size of 0.15 and an *α* of 0.05, the study power is 0.849. Post hoc power was calculated using G* power 3.0.

## Results

### Characteristics of the Study Sample

After a follow-up period of 14±1.6 months, 76 subjects underwent a second BMD assessment. Basic characteristics are summarized in Table 1 and 2. Among these patients, 12 had a medical history of DM; 10 were infected with HBV, 15 with HCV, and 13 with CMV; 10 men were habitual users of tobacco; and 8 men and 1 woman regularly consumed alcohol. Thirty-four patients received alendronate, 57 prednisolone, 55 mycophenolate, 30 tacrolimus, 26 cyclosporine, and 34 sirolimus. A single immunosuppressive agent was administered to 8 (11%) patients; 21 (28%) patients received 2 immunosuppressive agents, 36 (47%) received 3, and 11 (14%) received 4. Of the 42 non–alendronate users, 9 had HCV infection; and 6 of the 34 alendronate users had HCV infection (Table 3).

**Table 1:** Baseline characteristics of 76 patients.

Variable	n
**Male sex**	36
**Smoking**	10
**Alcohol**	9
**DM**	12
**HBV**	10
**HCV**	15
**CMV**	13
**Use of prednisolone**	57
**Use of mycophenolate**	55
**Use of tacrolimus**	30
**Use of cyclosporine**	26
**Use of sirolimus**	34
**Use of alendronate sodium**	34
**1 immunosuppressive agent**	8
**2 combined immunosuppressive agents**	21
**3 combined immunosuppressive agents**	36
**4 combined immunosuppressive agents**	11

Abbreviations: n, number of patients; CMV, cytomegalovirus; DM, diabetes mellitus; HBV, hepatitis B virus; HCV, hepatitis C virus.

**Table 2:** Laboratory results at baseline and at follow-up (N = 76).

Parameter	Baseline	Follow-up	*p*
**LS-BMD (g/cm^2^)**	0.90±0.14	0.92±0.14	<0.001
**H-BMD (g/cm^2^)**	0.81±0.14	0.81±0.14	NS
**FN-BMD (g/cm^2^)**	0.68±0.12	0.69±0.13	NS
**LS T**	−1.53±1.24	−1.32±1.26	<0.001
**H T**	−1.76±0.97	−1.68±1.07	NS
**FN T**	−2.45±0.96	−2.42±1.02	NS
**BUN (mg/dL)**	19.7±10.0	19.8±10.9	NS
**Cr (mg/dL)**	1.18±0.54	1.20±0.54	NS
**C-Ca (mg/dL)**	9.11±0.47	9.01±0.47	0.01
**P (mg/dL)**	3.21±0.54	3.23±0.59	NS
**Uric acid (mg/dL)**	6.24±1.68	6.45±1.70	NS
**Albumin (g/L)**	4.42±0.29	4.34±0.40	0.009
**TC (mg/dL)**	201±44	190±46	0.044
**TG (mg/dL)**	147±82	148±113	NS
**Normal BMD**	5	5	
**Osteopenia**	30	29	
**Osteoporosis**	41	42	

Abbreviations: BMD, bone mineral density; BUN, blood urea nitrogen; C-Ca, serum corrected calcium; CMV, cytomegalovirus; Cr, blood creatinine; DM, diabetes mellitus; HBV, hepatitis B virus; HCV, hepatitis C virus; FN, femoral neck; H hip; LS, lumbar spine; NS, not significant (*p*>0.05); P, serum inorganic phosphate; T, number of standard deviations above or below the mean value of BMD for sex-matched young adults; TC, serum total cholesterol; TG, serum triglyceride. LS-BMD was significantly greater at follow-up than at baseline. The albumin and calcium levels also decreased significantly, but they remained within normal ranges.

**Table 3:** Baseline and follow-up data for non–alendronate and alendronate patients.

	Non-alendronate (n = 42)			Alendronate (n = 34)			
Parameter	Baseline	Follow-up	*p*	Baseline	Follow-up	*p*	*P*
**LS-BMD (g/cm^2^)**	0.96±0.13	0.97±0.14	0.007	0.82±0.11	0.86±0.12	<0.001	0.002
**H-BMD (g/cm^2^)**	0.87±0.13	0.87±0.14	NS	0.72±0.10	0.73±0.10	NS	NS**^§^**
**FN-BMD (g/cm^2^)**	0.74±0.11	0.75±0.12	NS	0.60±0.05	0.60±0.06	NS	NS**^§^**
**LS T**	−1.01±1.15	−0.86±1.24	NS	−2.15±1.04	−1.87±1.06	<0.001	
**H T**	−1.27±0.80	−1.22±0.97	*NS*	−2.35±0.82	−2.24±0.9	NS	
**FN T**	−1.89±0.90	−1.86±0.99	*NS*	−3.12±0.44	−3.08±0.54	NS	
**Age (y)**	49±9.1			52.7±8.7			NS*
**Male sex**	22/42			14/34			NS*
**Smoking**	6/42			4/34			NS*
**Alcohol**	6/42			3/34			NS*
**DM**	10/42			2/34			NS*
**HBV**	4/42			6/34			NS*
**HCV**	9/42			6/34			NS*
**CMV**	7/42			6/34			NS*
**BUN (mg/dL)**	17.7±7.6	17.8±6.5	NS	21.94±12.33	22.1±14.3	NS	NS**^§^**
**Cr (mg/dL)**	1.08±0.41	1.14±0.38	0.034	1.27±0.64	1.31±0.67	NS	NS**^§^**
**C-Ca (mg/dL)**	9.13±0.46	9.04±0.49	NS	9.1±0.48	9.0±0.41	NS	NS**^§^**
**P (mg/dL)**	3.19±0.58	3.19±0.58	NS	3.23±0.49	3.27±0.6	NS	NS**^§^**
**Uric acid (mg/dL)**	5.94±1.63	6.5±1.70	0.036	6.64±1.74	6.36±1.64	NS	NS**^§^**
**Albumin (g/L)**	4.46±0.31	4.37±0.40	0.02	4.37±0.26	4.30±0.40	NS	NS**^§^**
**TC (mg/dL)**	194±40	185±50	NS	210±47	194±40	0.038	NS**^§^**
**TG (mg/dL)**	149±83	143±99	NS	146±82	154±128	NS	NS**^§^**
**Normal BMD**	5	5		0	0		
**Osteopenia**	30	25		0	4		
**Osteoporosis**	7	12		34	30		
**Use of prednisolone**	66.7%			85.3%			NS*
**Use of mycophenolate**	73.8%			70.6%			NS*
**Use of tacrolimus**	33.3%			47.1%			NS*
**Use of sirolimus**	52.4%			35.3%			NS*
**Use of cyclosporine**	33.3%			35.3%			NS*

Abbreviations: BMD, bone mineral density; BUN, blood urea nitrogen; C-Ca, serum corrected calcium; Cr, blood creatinine; CMV, cytomegalovirus; DM, diabetes mellitus; FN, femoral neck; H, hip; HBV, hepatitis B virus; HCV, hepatitis C virus; LS, lumbar spine; NS, not significant (*p*>0.05); P, serum inorganic phosphate; T, number of standard deviations above or below mean BMD of sex-matched young adults; TC, serum total cholesterol; TG, serum triglyceride. NS*, no significance (p>0.05) between non-alendronate patients and alendronage patients. NS**^§^**, no significance (p>0.05) of difference values between non-alendronate patients and alendronage patients. At follow-up, LS-BMD was significantly increased in the alendronate and non-alendronate groups (*p*<0.05). The albumin levels also decreased significantly in patients not taking alendronate sodium, but they remained within normal ranges. The uric acid and creatinine levels were significantly increased at follow-up in patients not taking alendronate sodium, but these also remained within normal ranges.

### Bone Mineral Density at Baseline and Follow-up

Table 3 presents the changes in BMD and blood biochemistry after 14±1.6 months of follow-up in patients who did and did not use alendronate. In the 42 non–alendronate users, serum albumin level decreased from 4.46±0.31 g/L to 4.37±0.40 g/L (*p* = 0.02). However, BMD of the lumbar spine increased from 0.96±0.13 g/cm^2^ to 0.97±0.14 g/cm^2^ (*p* = 0.007). The number of patients with osteoporosis increased from 7 to 12. In the 34 alendronate users, total cholesterol level decreased from 210±47 mg/dL to 194±40 mg/dL (*p* = 0.038), and BMD of the lumbar spine increased from 0.82±0.11 g/cm^2^ to 0.86±0.12 g/cm^2^ (*p*<0.001). The number with osteoporosis decreased from 34 to 30. There was no significant change in bone mineral density of the hip bone or femoral neck in either group. No correlation between use of alendronate and use of immunosuppressive agents was found with chi-square test (*p*>0.05).

### Factors Associated with Bone Turnover in the Presence or Absence of Alendronate

We performed subgroup analysis to clarify the difference between patients who did and did not take alendronate and showed the difference values in table 3. The alendronate users had greater increase in lumbar spine BMD than the non–alendronate patients (0.035 [0.016, 0.052] g/cm^2^ vs. 0.009 [−0.0032, 0.036] g/cm^2^, *p* = 0.002). The cumulative dose of prednisolone was significantly greater in the alendronate group (1450 mg ±984.5 mg vs. 735±699 mg, p = 0.001). Cumulative doses of other immunosuppressive agents were not different between alendronate patients and non–alendronate patients (*p*>0.05). Age (49 y ±9.1 y vs. 52.7 y ±8.7 y), male sex (52.4% vs. 41.2%), HCV infection (22.5% vs. 18.8%), HBV infection (9.8% vs. 17.6%), CMV infection (17.5% vs. 18.8%), creatinine change (0.005 [−0.05, 0.142] mg/dL vs. 0.015 [−0.082, 0.162] mg/dL), serum albumin change (−0.06 [−0.225, 0.075] g/L vs. −0.02 [−0.215, 0.15] g/L), hip bone BMD change (−0.0005 [−0.021, 0.019] g/cm^2^ vs. 0.018 [−0.011, 0.034] g/cm^2^), and femoral neck BMD change (0 [−0.022, 0.021] g/cm^2^ vs. 0.005 [−0.022, 0.021] g/cm^2^) were not significantly different (*p*>0.05) between alendronate and non-alendronate patients.

### Factors Associated with Bone Turnover in the 76 Patients

In order to investigate the clinical features on bone condition, we used simple linear regression to evaluate associations between BMD change in the lumbar spine, hip bone, and femoral neck and the other clinical variables in 76 patients (table 4). Use of alendronate (***β***: 0.345, 95% CI, 0.007 to 0.033; *p* = 0.002) and HCV infection (***β***: −0.262, 95% CI, −0.037 to −0.002; *p = *0.027) were associated in patients with BMD change in the lumbar spine. Time since transplantation (***β***: −0.275, 95% CI, −0.0002 to −0.0001; *p* = 0.018) was associated in patients with BMD change in Hip bone. Age (***β***: −0.423, 95% CI, −0.003 to −0.001; *p*<0.001) and age at transplant (***β***: −0.373, 95% CI, −0.003 to −0.001, *p* = 0.001) were associated in patients with BMD change in the femoral neck. In advanced multiple linear regression in 76 patients, HCV infection (***β***: −0.247, 95% CI, −0.035 to −0.002, *p* = 0.028) and use of alendronate (***β***: 0.37, 95% CI, 0.009 to 0.036, *p* = 0.001) were the significant risk factors with BMD change of lumbar spine; time since transplantation (***β***: −0.293, 95% CI, −0.0002 to −0.0001, *p* = 0.011) and use of alendronate (***β***: 0.246, 95% CI, 0.001 to 0.033, *p* = 0.035) were the significant risk factors with BMD change of hip bone and age (***β***: −0.464, 95% CI, −0.003 to −0.001, *p*<0.001) was the significant risk factor with BMD change of femoral neck.

**Table 4:** Clinical variables associated with bone mineral density change (N = 76).

	Lumbar Spine				Hip Bone				Femoral Neck			
Variable	Simple *β* (95% CI)	*p*	Multiple *β* (95% CI)	*p*	Simple *β* (95% CI)	*p*	Multiple *β* (95% CI)	*p*	Simple *β* (95% CI)	*p*	Multiple *β*(95% CI)	*p*
**Age**	0.16(−0.001, 0.001)	NS			−0.072(−0.001, 0.001)	NS			−0.423(−0.003, −0.001)	<0.001	−0.464(−0.003, −0.001)	<0.001
**Male sex**	0.093 (−0.008, 0.019)	NS			0.0001(−0.016, 0.016)	NS			0.15(−0.007, 0.032)	NS		
**DM**	−0.067 (−0.025, 0.016)	NS			−0.213(−0.041, 0.002)	NS			−0.014(−0.029, 0.026)	NS		
**HBV**	0.14(−0.008, 0.032)	NS			0.018(−0.022, 0.025)	NS			−0.027(−0.032, 0.026)	NS		
**HCV**	−0.262(−0.037, −0.002)	0.027	−0.247(−0.035, −0.002)	0.028	−0.197(−0.037, 0.003)	NS			−0.018(−0.028, 0.024)	NS		
**CMV**	−0.102(−0.026,0.011)	NS			−0.025(−0.023, 0.019)	NS			−0.056(−0.033, 0.020	NS		
**Smoking**	0.043(−0.017, 0.024)	NS			−0.054(−0.029, 0.018)	NS			−0.023(−0.032, 0.027)	NS		
**Alcohol**	−0.023(−0.023, 0.019)	NS			0.039(−0.021, 0.029)	NS			0.066(−0.022, 0.039)	NS		
**Time since transplantation**	−0.061(−0.001,0.001)	NS			−0.275(−0.0002, −0.0001)	0.018	−0.293(−0.0002, −0.0001)	0.011	−0.037(−0.001, 0.0001)	NS		
**Age at transplantation**	0.198(−0.001,0.001)	NS			0.078(−0.001,0.001)	NS			−0.373(−0.003, −0.001)	0.001		
**Prednisolone**	0.085(−0.01, 0.021)	NS			−0.012(−0.019, 0.017)	NS			−0.036(−0.026, 0.019)	NS		
**Mycophenolate**	−0.028(−0.017, 0.013)	NS			−0.021(−0.019, 0.016)	NS			0.07(−0.015, 0.028)	NS		
**Tacrolimus**	0.138(−0.006, 0.022)	NS			0.163(−0.005, 0.027)	NS			−0.075(−0.027, 0.014)	NS		
**Sirolimus**	−0.055(−0.017, 0.01)	NS			0.047(−0.013, 0.019)	NS			0.098(−0.011, 0.028)	NS		
**Cyclosporine**	−0.081(−0.02, 0.009)	NS			−0.132(−0.026, 0.007)	NS			−0.05(−0.025, 0.016)	NS		
**Alendronate**	0.345(0.007, 0.033)	0.002	0.37 (0.009, 0.036)	0.001	0.197(−0.002, 0.029)	NS	0.246 (0.001, 0.033)	0.035	−0.01(−0.021, 0.019)	NS		

Abbreviations: CMV, cytomegalovirus; DM, diabetes mellitus; HBV, hepatitis B virus; HCV, hepatitis C virus; NS, not significant (*p*>0.05). Initially, simple linear regression analysis was performed to identify the variables associated with bone mineral density (BMD) change in 76 patients. HCV infection and use of alendronate were associated with BMD change of the lumbar spine. Time since transplantation and use of alendronate were associated with BMD change of the hip bone. Age and age at transplantation were associated with BMD change of the femoral neck. In advanced analysis by multiple linear regression, HCV infection was still the risk factor for adverse BMD change of the lumbar spine. ***β*** (95% CI): Linear regression standardized coefficients (95% confidence intervals).

From the result of table 4, HCV infection was the significant risk factors with BMD change of lumbar spine but not in hip bone and femoral neck. To further our investigation of the influence of clinical features on bone condition between without and with alendronate patients, we used simple linear regression to evaluate associations between BMD change in the lumbar spine and the other clinical variables in the alendronate and non- alendronate groups (table 5). In non–alendronate patients (n = 42), HCV infection was associated in patients with BMD change in the lumbar spine (***β***: −0.36, 95% CI, −0.043 to −0.003; *p* = 0.023). In patients taking alendronate sodium (n = 34), age at transplantation (***β***: 0.379, 95% CI, 0–0.002, *p* = 0.03) showed an association in patients with BMD change in the lumbar spine. In advanced multiple linear regression, in non–alendronate patients (n = 42), HCV infection was the significant risk factor with BMD change of lumbar spine (***β***: −0.371, 95% CI, −0.044 to −0.004, *p* = 0.023). Otherwise, in patients with alendronate sodium (n = 34), no clinical was the significant risk factor with BMD change of lumbar spine.

**Table 5:** Clinical variables associated with bone mineral density change of the lumbar spine in patients taking (n = 34) and not taking (n = 42) alendronate sodium.

	No Alendronate				Alendronate			
Variable	Simple *β* (95% CI)	*p*	Multiple *β* (95% CI)	*p*	Simple *β* (95% CI)	*p*	Multiple *β* (95% CI)	*p*
**Age**	−0.072(−0.001, 0.001)	NS	−0.004	NS	0.276(0, 0.002)	NS	−	−
**Male sex**	0.158(−0.009, 0.026)	NS	0.118	NS	0.16(−0.011, 0.029)	NS	−	−
**DM**	−0.067 (−0.025, 0.016)	NS	−0.149	NS	−0.138(−0.058, 0.026)	NS	−	−
**HBV**	0.21(−0.01, 0.047)	NS	0.218	NS	−0.006(−0.027, 0.026)	NS	−	−
**HCV**	−0.36 (−0.043, −0.003)	0.023	−0.371 (−0.043, −0.003)	0.023	−0.043 (−0.03, 0.024)	NS	−	−
**CMV**	0.013 (−0.022, 0.024)	NS	0.087	NS	−0.285(−0.046, 0.005)	NS	−	−
**Smoking**	0.081 (−0.019, 0.031)	NS	0.148	NS	0.016(−0.03, 0.033)	NS	−	−
**Alcohol**	0.081(−0.019, 0.031)	NS	0.148	NS	−0.118(−0.047, 0.024)	NS	−	−
**Time since transplantation**	−0.055(−0.0001, 0.0001)	NS	0.074	NS	−0.237(−0.0001, 0.0001)	NS	−	−
**Age at transplantation**	−0.023(−0.001, 0.001)	NS	−0.038	NS	0.379(0, 0.002)	0.03	−	−
**Prednisolone**	0.165(−0.009, 0.028)	NS	0.157	NS	−0.219(−0.045, 0.01)	NS	−	−
**Mycophenolate**	0.004 (−0.02, 0.02)	NS	−0.097	NS	−0.017 (−0.023, 0.021)	NS	−	−
**Tacrolimus**	0.018 (−0.018, 0.02)	NS	0.021	NS	0.151(−0.011, 0.028)	NS	−	−
**Sirolimus**	0.093 (−0.012, 0.023)	NS	−0.052	NS	−0.138(−0.029, 0.013)	NS	−	−
**Cyclosporine**	−0.178 (−0.029, 0.008)	NS	−0.034	NS	0.06(−0.017, 0.024)	NS	−	−

Abbreviations: CMV, cytomegalovirus infection; DM, diabetes mellitus; HBV, hepatitis B virus infection; HCV, hepatitis C virus infection; NS, not significant (*p*>0.05). Initially, simple linear regression analysis was performed to identify the variables associated with BMD change in patients taking alendronate sodium and those who were not. HCV infection was negatively associated with BMD change of the lumbar spine in patients not taking alendronate. Age at transplantation was positively associated with BMD change in the lumbar spine in patients taking alendronate. In advanced analysis by multiple linear regression, HCV infection was still the risk factor for adverse BMD change of the lumbar spine in patients without alendronate. No clinical variable was a risk factor for predicting BMD change of the lumbar spine in patients taking alendronate. ***β*** (95% CI): Linear regression standardized coefficients (95% confidence intervals).

## Discussion

In this study, we showed that HCV infection was a risk factor for adverse BMD change in renal transplantation patients, especially in the lumbar spine. The negative effect of BMD change associated with HCV infection could be eliminated by using alendronate.

HCV infection is still highly prevalent among renal transplant recipients in both developed and developing countries [1], [2]. The natural history of HCV infection after renal transplantation is still unclear [1]. Most studies of HCV infection in renal transplant recipients [17]–[19] have focused on mortality, graft survival, treatment response, and liver condition. Discussion of the correlation between HCV infection and bone condition in renal transplant recipients is limited. In this study, we showed that HCV infection was a risk factor for BMD change, especially in the lumbar spine, in renal transplant recipients.

It is well known that in renal transplant recipients, increase in bone mass loss is multifactorial and is affected by age [20], sex [20]–[22], renal function, and duration of dialysis before transplantation [22]. A major, well-known cause of increased loss of bone mass is high-dose steroid therapy during the early period after transplantation and long-term steroid administration [23]. In a previous cross-sectional study [13], renal transplant recipients with HCV infection had high prevalence rates of osteoporosis and osteopenia. In the general population, lower bone mineral density has been associated with chronic liver disease, including chronic viral hepatitis [8], but there was no correlation between severity of liver disease and bone density [24]. In the Mayo Clinic studies of BMD in more than 200 patients with advanced cirrhosis, 37% of the HCV-positive patients had osteopenia and 28% had osteoporosis. The percentage with osteoporosis was significantly higher in the HCV group than in the alcoholic liver disease (ALD) group, whereas the ALD group had more-advanced liver disease [25]. Another study comparing patients with HCV cirrhosis to those with ALD cirrhosis found that all patients with HCV infection had low bone density, while only half of the ALD group did [26].

Regarding osteoporosis caused by HCV, recent studies have focused on HIV/HCV co-infection [11], [12], . Osteoporosis was very common in African-American patients with HIV/HCV co-infection [28]. Hansen et al. [27] also pointed out that the relative risk of fracture was higher in HIV/HCV co-infection patients than in HIV monoinfection patients. To our knowledge, HIV is the cause of a spectrum of disease known as HIV/AIDS. HIV is a retrovirus that primarily infects components of the human immune system, such as CD4^+^ T cells, macrophages, and dendritic cells. It directly and indirectly destroys CD4^+^ T cells [29]. The mechanism by which HIV/HCV co-infection aggravates BMD change is still not clearly understood. Immunosuppressive agents prescribed for transplant recipients affect T-cell activation and subsequent cellular proliferation. These findings indicate that patients with HIV and transplant recipients taking immunosuppressive agents have a similar condition. To our knowledge, studies of bone condition in renal transplant recipients with HCV infection are rare. It seems that HCV infection in the presence of suppressed T-cell activity may have a role in BMD loss.

Recently, a cytokine system (including receptor activator of nuclear factor kappa-B [RANK], receptor activator of nuclear factor kappa-B ligand [RANKL], and osteoprotegerin [OPG]) [30]–[32] was found to regulate the proliferation, differentiation, fusion activation, and apoptosis of osteoclasts [33]–[35]. Several tissues produce RANKL, especially lymphoid tissues and bone [30]–[32], [36], [37]. Osteoporosis caused by portosystemic shunting [38], or by steroid or CIs through receptor activator of RANKL-dependent pathways, may be partially ameliorated using sirolimus [39]. Recently, serum levels of soluble RANKL were measured in patients with chronic liver disease. Szalay et al. [40] showed that patients with primary biliary cirrhosis tended to have high OPG and low RANKL levels, and patients with chronic HCV infection exhibited high OPG and RANKL levels. Moschen et al. [41] demonstrated high OPG and RANKL levels in patients with liver disease but without cirrhosis, including those with alcoholic hepatitis, primary biliary cirrhosis, and viral hepatitis. Another study [42] showed that patients with alcoholic liver cirrhosis had high OPG and RANKL levels.

It is worth to note that in our study, HCV infection had a negative role of BMD change of the lumbar spine. However, in hip bone and femoral neck, the role of HCV infection was not found. In our knowledge, vertebrae consist of about 50% trabecular bone, while other bones (hip, femoral neck and trochanter) consist mainly of cortical bone [43]. In clinical study by George el al [26], among the 79 chronic liver disease patients, 49 had low BMD and lumbar spine was the most frequently and severely affected site. All of the patients with HCV infection had low BMD. In addition, in a study on osteoporosis in patients with end- stage liver disease caused by hepatitis C and ALD, Carey et al. [25] showed that hepatitis C patients had the lowest lumbar bone density and ALD patients had the highest bone density. In a prospective cohort study [28], El-Maouche et al. showed that osteoporosis was very common in the lumbar spine among the African-American patients with HIV/HCV co-infection. It is unclear about the mechanism between HCV infection and bone loss of lumbar spine. However, from above cited references, it may be explained that sites with a high proportion of trabecular bone are affected earliest in diseases like HCV infection that produce increased bone turnover.

Previously, vitamin D status was assessed in patients with noncholestatic chronic liver diseases [44]. Some studies [45], [46] showed that HCV infection may reduce vitamin D levels. About 70% of non-cirrhotic HCV-positive patients had 25(OH)D deficiency (<20 ng/mL), and 14% had 25(OH)D severe deficiency (<7 ng/mL) [44]. Vitamin D deficiency, secondary hyperparathyroidism, and osteoporosis often coexist in elderly women [47]–[49]. In a randomized, controlled study, Barone et al. pointed out that elderly women with osteoporosis caused due to hypovitaminosis D had a good response to alendronate [50]. Another study found no significant difference in BMD response to treatment in vitamin D–replete compared to vitamin D–deficient postmenopausal women who were taking bisphosphonates [51]. Based on these references, we can explain why HCV was not a risk factor for BMD change in our 34 alendronate patients. Further, it seems that alendronate may be able to reverse bone lesions in renal transplant recipients with HCV infection.

Although the limitations of our study (its retrospective design and small sample) are evident, to our knowledge, these are the first reported data showing the effect of HCV infection on bone in renal transplant patients and indicating that alendronate can counteract this negative effect.

### Conclusion

This randomized case-control study has shown that HCV infection in renal transplant recipients was a risk factor for adverse BMD change of the lumbar spine. Moreover, alendronate may be able to reverse bone lesions in renal transplant recipients with HCV infection.
